# RAFT Emulsion Polymerization of Styrene Using a Poly((*N,N*-dimethyl acrylamide)-*co*-(*N*-isopropyl acrylamide)) mCTA: Synthesis and Thermosensitivity

**DOI:** 10.3390/polym14010062

**Published:** 2021-12-24

**Authors:** Katharina Nieswandt, Prokopios Georgopanos, Martin Held, Evgeni Sperling, Volker Abetz

**Affiliations:** 1 Helmholtz-Zentrum Hereon, Institute of Membrane Research, Max-Planck-Straße 1, 21502 Geesthacht, Germany; katharina.nieswandt@hereon.de (K.N.); martin.held@hereon.de (M.H.); evgeni.sperling@hereon.de (E.S.); 2 Institute of Physical Chemistry, University of Hamburg, Martin-Luther-King-Platz 6, 20146 Hamburg, Germany

**Keywords:** RAFT, emulsion polymerization, PISA, random copolymers, block copolymers, micelles, stimuli-responsive, thermo-responsive, LCST

## Abstract

Thermoresponsive poly((*N,N*-dimethyl acrylamide)-*co*-(*N*-isopropyl acrylamide)) (P(DMA-*co*-NIPAM)) copolymers were synthesized via reversible addition−fragmentation chain transfer (RAFT) polymerization. The monomer reactivity ratios were determined by the Kelen–Tüdős method to be *r*_NIPAM_ = 0.83 and *r*_DMA_ = 1.10. The thermoresponsive properties of these copo-lymers with varying molecular weights were characterized by visual turbidimetry and dynamic light scattering (DLS). The copolymers showed a lower critical solution temperature (LCST) in water with a dependence on the molar fraction of DMA in the copolymer. Chaotropic and kosmotropic salt anions of the Hofmeister series, known to affect the LCST of thermoresponsive polymers, were used as additives in the aqueous copolymer solutions and their influence on the LCST was demonstrated. Further on, in order to investigate the thermoresponsive behavior of P(DMA-*co*-NIPAM) in a confined state, P(DMA-*co*-NIPAM)-*b*-PS diblock copolymers were prepared via polymerization induced self-assembly (PISA) through surfactant-free RAFT mediated emulsion polymerization of styrene using P(DMA-*co*-NIPAM) as the macromolecular chain transfer agent (mCTA) of the polymerization. As confirmed by cryogenic transmission electron microscopy (cryoTEM), this approach yielded stabilized spherical micelles in aqueous dispersions where the PS block formed the hydrophobic core and the P(DMA-*co*-NIPAM) block formed the hydrophilic corona of the spherical micelle. The temperature-dependent behavior of the LCST-type diblock copolymers was further studied by examining the collapse of the P(DMA-*co*-NIPAM) minor block of the P(DMA-*co*-NIPAM)-*b*-PS diblock copolymers as a function of temperature in aqueous solution. The nanospheres were found to be thermosensitive by changing their hydrodynamic radii almost linearly as a function of temperature between 25 °C and 45 °C. The addition of kosmotropic salt anions, as a potentially useful tuning feature of micellar assemblies, was found to increase the hydrodynamic radius of the micelles and resulted in a faster collapse of the micelle corona upon heating.

## 1. Introduction

Over the past decade, stimuli-responsive “smart” polymers, capable of tuning their physicochemical properties and structural conformations, have been intensively studied [1,2]. These materials can change their properties and are triggered by a change in the materials’ environment or the modification of certain external stimuli such as temperature, pH, electric potential, salt concentration, pressure, light, or a magnetic field [3,4,5,6,7]. Of particular interest are temperature-responsive polymers that show a lower critical solution temperature (LCST) [8]. These reversible and entropically controlled phase transitions are usually manifested by a rapid change of the polymers’ solubility in water [9,10,11].

Despite enormous efforts, there are still no universal theories that delineate the molecular processes associated with stimuli-responsive solubility. However, it is believed that this behavior is mainly dictated by solvent-polymer interactions [12] and hydrogen bonding between the water molecules and the hydrophilic polymer side chains [13]. Below the LCST, these interactions are energetically favored but result in entropy loss since only specific angles between the water and solute allow hydrogen bond formation [14]. The polymer chains collapse as soon as the temperature surpasses the so-called cloud point or phase transition temperature (PTT) due to the breaking of hydrogen bonds and hydrophobic polymer−polymer interactions. The entropy component outweighs the enthalpy component and the dissolved state of the polymer becomes thermodynamically unfavorable. On a macroscopic level, the polymer precipitates, while on a molecular level, the process is described as ‘coil-to-globule transition’ [15,16].

Stimuli-responsive polymers are high-performance “smart” materials as they can be employed as switchable filtration devices [17,18]. In recent years, novel stimuli-responsive membranes have received enormous attention [19]. Their stimuli-responsive properties can be tailored during synthesis by varying the chemical composition, chain length, and molecular architecture of the stimuli-responsive membrane materials or post-synthetical modification after membrane fabrication [19]. Additionally, other potential fields of application are essential components in technical devices such as sensors or prospective drug delivery systems [19,20,21]. The researchers primarily focus on the self-assembly of amphiphilic block copolymers, consisting of a hydrophilic thermoresponsive block and a suitable hydrophobic block. The low critical micelle concentration and the associated high stability of the micelles make amphiphilic block copolymers ideal systems for already established, potential applications in the synthesis of polymers via emulsion polymerization, colloid stabilization and continuous flow chemistry [22,23]. Especially the thermoresponsive formation of micelles or vesicular structures in aqueous solutions of poly(*N*-isopropyl acrylamide) (PNIPAM) and its block copolymers have been some of the most extensively studied structures. Due to its LCST of 32 °C close to the physiological temperature, PNIPAM has long been considered the “gold standard” of thermoresponsive polymers [24,25]. Statistical copolymerization with other thermoresponsive or unrestricted water-soluble/hydrophilic monomers even allows the LCST to be raised to precisely the physiological temperature or above, making these systems additionally attractive for use in drug delivery systems [25,26,27,28]. However, for a sharp transition, a random copolymerization of certain thermoresponsive building units and thus the avoidance of a compositional drift along the chain are beneficial [29]. The use of chemically similar co-monomers with similar reactivity towards radicals is, therefore, advantageous [30]. To date, several hydrophobic segments in the amphiphilic LCST-type diblock copolymers were investigated [31,32,33,34,35,36]. In continuation of the work of Eggers et al. [33] and Lauterbach et al. [37], who investigated the thermoresponsive behavior of amphiphilic poly(*N*-acryloyl pyrrolidine)-*b*-polystyrene and poly(*N,N*-dimethyl acrylamide)-*b*-poly(*N*-acryloyl piperidine-*co*-*N*-acryloyl pyrrolidine)-*b*-polystyrene block copolymers, the present work also used polystyrene as a hydrophobic block, which makes the block copolymer water-insoluble.

For decades, the well-controlled radical polymerization of slowly-propagating radicals, such as resonance-stabilized styrenics, appeared challenging [33]. Back then, polystyrene latexes were produced mainly through free radical heterogeneous polymerizations stabilized and controlled by surfactants as emulsifiers [33,37,38,39]. Nowadays, research and industrially relevant (block) copolymers with given molecular weights and architectures can be prepared by combining controlled reversible deactivation radical polymerization (RDRP) with emulsion or dispersion polymerization techniques [37,40]. RDRP techniques have received enormous attention from scientists all over the world. The RDRP techniques include the extensively studied atom transfer radical polymerization (ATRP), nitroxide-mediated polymerization (NMP) and reversible addition-fragmentation chain transfer (RAFT) polymerization. Especially RAFT emulsion and dispersion polymerization techniques have become a powerful tool for the efficient synthesis of the diblock copolymer. They provide an efficient heat transfer, a low viscosity during the entire reaction time and enable the production of well-defined polymers with high molar masses and narrow dispersity [37,41,42,43,44,45]. RAFT-mediated polymerization-induced self-assembly (PISA) has been studied thoroughly by a variety of scientific groups such as Armes et al. [46], Troung et al. [47], and Boyer et al. [48], to name a few, effectively advancing the research in this area. PISA occurs through chain extension of the solvophilic macromolecular chain transfer agent (mCTA) with a solvophobic block, as the latter becomes insoluble above a certain block length, resulting in micellization [49,50,51,52]. In the case of emulsion polymerization, the chain growth before micellar nucleation is slow and depends (stage 1) on the diffusion of the solvophobic monomer molecules from the monomer droplets into the solvent phase. The polymerization rate increases massively after micellization (stage 2). The RAFT emulsion polymerization of styrene has been studied using thermo- and pH-responsive mCTAs [33,37,44].

P(DMA-*co*-NIPAM) copolymers have been prepared successfully by Liu et al. [23,53,54], Barker et al. [55] and others [56,57,58,59,60] via conventional radical polymerization and by Bauri et al. via RAFT polymerization [61]. Over the past two decades, the thermoresponsive properties of the synthesized copolymers have been intensively studied by these groups. For example, Barker et al. determined optically visible cloud points of the copolymers as a function of the DMA content in the copolymer. Bauri and co-workers performed differential scanning calorimetry and thermogravimetric analyses at different heating rates on these copolymers to understand their thermal degradation in the solid state. Nevertheless, other influences on the LCST, for example the influence of additives on the cloud points of the aqueous copolymer solutions were not exhaustively studied.

In the present study thermoresponsive trithiocarbonate terminated P(DMA-*co*-NIPAM) copolymers, synthesized via RAFT solution polymerization, were employed as mCTA in the surfactant-free RAFT emulsion polymerization of styrene at 70 °C in water/1,4-dioxane. in order to gain a more profound knowledge of the thermoresponsive behavior of the P(DMA-*co*-NIPAM) and P(DMA-*co*-NIPAM)-*b*-PS copolymers, a variety of copolymers were synthesized and their properties were investigated in aqueous solution. in addition, chaotropic and kosmotropic salts with ions of the Hofmeister series, which are known to influence the LCST of thermoresponsive polymers, were added to the aqueous copolymer solutions and their influence on the solution behavior was investigated. Since the study at hand addresses questions regarding the impact of the molecular architecture of thermoresponsive polymers and critical parameters (e.g., solvent system, temperature) on the self-assembly, swelling and other physicochemical properties of polymers in solutions, the diblock copolymers were investigated with respect to their film formation behavior by dynamic light scattering (DLS) as well as transmission electron microscopy at cryogenic conditions (cryoTEM) of the emulsions. Their bulk morphologies were analyzed via transmission electron microscopy (TEM).

## 2. Materials and Methods

2,2′-Azobis(2-methylpropionitrile) (AIBN, 98%, Sigma-Aldrich, Taufkirchen, Germany, stored at 4 °C), 4,4′-Azobis(4-cyanovaleric acid (ACVA, ≥98%, Sigma-Aldrich, Taufkirchen, Germany, stored at 4 °C) 4-cyano-4-[(dodecylsulfanylthiocarbonyl)sulfanyl]pentanoic acid (CDTPA, 97%, Sigma-Aldrich, Taufkirchen, Germany, stored at 4 °C), *N*-ispropoyl acrylamide (NIPAM, ≥99%, Sigma-Aldrich, Taufkirchen, Germany, stored at 4 °C), chloroform (CHCl_3_, ≥99.8%, VWR, Bruchsal, Germany), chloroform-*d*_1_ (CDCl_3_, 99.8%, contains 0.03% (*v/v*) TMS, Sigma-Aldrich, Taufkirchen, Germany), diethyl ether (≥99.5%, Sigma-Aldrich, Taufkirchen, Germany), *N,N*-dimethyl acetamide (DMAc, ≥99.9%, Sigma-Aldrich, Taufkirchen, Germany), tetrahydrofuran (THF, 99.8%, Merck, Darmstadt, Germany), tetrahydrofuran-*d*_8_ (99.5%, Eurisotop, Saarbrücken, Germany) 1,4-dioxane (DOX, ≥99.5%, Sigma-Aldrich, Taufkirchen, Germany), *N,N*-dimethyl formamide (DMF, >99.5%, Merck, Darmstadt, Germany), sodium chloride (NaCl, ≥99.0%, Sigma-Aldrich, Taufkirchen, Germany), sodium bromide (NaBr, ≥99.0%, Sigma-Aldrich, Taufkirchen, Germany), sodium iodide (NaI, ≥99.0%, Sigma-Aldrich, Taufkirchen, Germany), sodium thiocyanate (NaSCN, ≥98.0% Sigma-Aldrich, Taufkirchen, Germany), ruthenium tetroxide (RuO_4_, 99.9%, Sigma-Aldrich, Taufkirchen, Germany) were used without further treatment and purification. Ultrapure MILLI-Q^®^ water (resistivity >18.2 MΩ∙cm^−1^) was obtained from a Millipore (Merck, Darmstadt, Germany) Direct-Q^®^ UV water purification system. Styrene (≥99%, Sigma-Aldrich, contained methyl ether hydroquinone as an inhibitor, stored at 4 °C) and *N,N*-dimethyl acrylamide (DMA, 99.5%, Alfar Aesar, Kandel, Germany, stabilized with 4-methoxyphenol, stored at 4 °C) were freshly percolated through a column of basic aluminum oxide (>98%, Sigma-Aldrich, Taufkirchen, Germany) to remove the inhibitor before use.

### 2.1. Synthesis of P(DMA-co-NIPAM) mCTA by RAFT Solution Copolymerization of DMA and NIPAM at 70 °C

A typical protocol for the synthesis of a P(DMA-*co*-NIPAM) mCTA is described below. DMA (360 mg, 3.6 mmol, 30 eq), NIPAM (2.64 g, 23.3 mmol, 191 eq), CDTPA RAFT agent (49.2 mg, 122 µmol, 1 eq), AIBN (2 mg, 12 µmol, 0.1 eq), 0.5 mL DMF (as an internal standard) and dioxane (8.9 mL, 25% (*w/w*)) were weighed into a 20 mL reaction vial ([NIPAM]/[DMA]/[CDTPA]/[AIBN] = 191/30/1/0.1). The solution was degassed by purging with nitrogen in an ice-water bath for 15 min. The polymerization was conducted at 70 °C and 850 rpm for 4 h and quenched by ice-cooling and air exposure. THF was added to the reaction mixture and the crude polymer was precipitated twice into diethyl ether. The polymer was dried under vacuum for 24 h and obtained as a light-yellow powder. The conversion of DMA and NIPAM amounted to 98.5% and 94.0%, respectively, as judged by ^1^H NMR spectroscopy. The corresponding ^1^H NMR spectrum is shown in Figure S1. GPC analysis indicated an apparent number average molecular weight (${\bar{M}}_{n,\mathrm{app}}$) of 39 kDa and a dispersity *Ð* of 1.09, were is the weight average molecular weight.

### 2.2. Synthesis of P(DMA-co-NIPAM)-b-PS via RAFT Emulsion Polymerization of Styrene at 70 °C

In a typical RAFT emulsion polymerization of styrene, the P(DMA-*co*-NIPAM) mCTA (336 mg, 11 µmol, 1 eq) was dissolved in H_2_O/1,4-dioxane (7.66 mL, 80:20, 20% *w/w*). ACVA (0.3 mg, 1 µmol, 0.1 eq) dissolved in 1,4-dioxane (100 µL) and 0.5 mL DMF (as an internal standard) were added to the solution. Styrene (1.59 g, 15 mmol, 1427 eq) was added ([styrene]/[P(DMA-*co*-NIPAM)]/[ACVA] = 1427/1/0.1) and the mixture was degassed by purging with nitrogen in an ice-water bath for 15 min. The polymerization was conducted at 70 °C and 850 rpm for 24 h and quenched by ice-cooling and air exposure. The crude polymer was isolated by removing water, 1,4-dioxane and DMF under reduced pressure. The crude polymer was dissolved in THF and poured into an excess of ice-cold *n*-hexane. The polymer was dried under vacuum at 40 °C for 24 h and obtained as a whitish powder. The conversion amounted to 79.0%, as calculated by ^1^H NMR spectroscopy. The corresponding ^1^H NMR spectrum is shown in Figure S1. GPC analysis indicated an of 85 kDa and a *Ð* of 1.50.

### 2.3. Characterization

#### 2.3.1. ^1^H NMR Spectroscopy

^1^H NMR spectroscopy experiments were performed using a Bruker AV500 spectro-meter. ^1^H NMR spectra were recorded applying a 10 ms 90° pulse at a sample temperature of 298 K. 16 scans were recorded with a relaxation delay of 3 s. Sample concentrations were 20 g∙L^−1^ in THF-d_8_ or CDCl_3_, respectively and the NMR spectra were analyzed with the software MestReNova 10.0 (Mestrelab Research, S.L., Spain).

The conversion of DMA and NIPAM was determined in CDCl_3_ from the decrease of the integrals of the monomer peaks at 5.70–5.65 ppm and 5.62–5.53 ppm, respectively, using the (CH_3_)_2_NC(O)H-peak (DMF) at 8.02 ppm as an internal standard. A reference sample was taken prior to and at the end of the polymerization. The conversion of styrene in the emulsion polymerization, determined in THF-*d*_8_, was calculated from the decrease of the integral of the monomer peaks at 6.08–5.64 ppm and 5.50–5.05 ppm employing again the (CH_3_)_2_NC(O)H-peak (DMF) at 8.02 ppm as an internal standard.

#### 2.3.2. Gel Permeation Chromatography (GPC)

The apparent molecular weights of the polymers were obtained by DMAc GPC containing lithium chloride (0.1 M) at a flow rate of 1.0 mL min^−1^ using a VWR-Hitachi L2130 pump (VWR Hitachi, Darmstadt, Germany) and a VWR-Hitachi L2490 RI (refractive index) detector (VWR Hitachi, Darmstadt, Germany). A Waters 717 plus instrument (Waters, Milford, MA, USA) equipped with PSS GRAM columns [GRAM pre-column (dimension 8–50 mm) and two GRAM columns of different porosity (3000 Å and 1000 Å)] with dimensions of 8 × 300 mm and a particle size of 10 μm was employed. Near-monodisperse polystyrene standards were used for calibration and the data were analyzed using the software PSS WinGPC UniChrom 8.4 (PSS, Mainz, Germany).

#### 2.3.3. Transmission Electron Microscopy (TEM) on Polymer Films

The bulk morphology of the diblock copolymers was investigated via TEM using a Tecnai G2 F20 electron microscope (Thermo Fisher Scientific, Amsterdam, The Netherlands), operating at an accelerating voltage of 120 kV in bright field mode. Polymer films were cast from solutions in CHCl_3_ and slowly dried in the presence of solvent vapor in a desiccator for 2 weeks. The films were further annealed thermally, stepwise, up to 155 °C in vacuum. ultrathin sections of approximately 100 nm were cut dry with a Leica Ultramicrotome EM UCT (Leica Microsystems, Wetzlar, Germany) equipped with a diamond knife (Diatome AG, Biel, Switzerland). Dry-sectioning was chosen since P(DMA-*co*-NIPAM) is water-soluble and would swell significantly if cut wet, disturbing the equilibrium of the copolymer structure. The polystyrene phase was selectively stained in RuO_4_-vapor for 10 min.

#### 2.3.4. Transmission Electron Microscopy on Polymerization Dispersions under Cryogenic Conditions (cryoTEM)

TEM images of the emulsion samples were recorded with the same electron microscope and the use of a cryo sample holder under cryogenic conditions in bright field mode, at an accelerating voltage of 120 kV. The samples were directly withdrawn from the dispersions after polymerization and diluted with MILLI-Q^®^ water or 0.5 M salt solutions (aqueous 0.5 M NaBr or NaSCN solution), respectively, to obtain a final polymer concentration of 2 mg/mL. For cryoTEM images, C-flat TEM grids with equidistant 1.2 µm holes were used (Protochips, Morrisville, NC, USA). These grids had previously been treated with H_2_/O_2_ plasma for 30 s in a Solarus 950 apparatus (Gatan, Pleasanton, CA, USA) to increase the wetting with an aqueous liquid. A plasma-treated grid was fixated with thin tweezers within a Cryo-plunge 3 system (Gatan, Pleasanton, CA, USA) and successively covered with 2 µL of diluted polymer dispersion, blotted for 2 s on both sides with filter paper and then plunged into liquid ethane with a temperature of −175 °C. The high cooling rate led to amorphously frozen ice. The vitrified samples were transferred under protection of liquid nitrogen into the cryo holder for electron microscopy. Non-careful warming of the samples would lead to crystallization of the ice, which would obscure the observation. For the same reason, the frozen samples were investigated under a low electron intensity to minimize warming effects.

#### 2.3.5. Transmission Electron Microscopy on Polymerization Dispersions at Room-Temperature

TEM images of the emulsion samples at ambient conditions were recorded as with the bulk polymer films. Two different preparation methods were used: for preparation method 1, the samples were directly withdrawn from the dispersions after polymerization and diluted with MILLI-Q^®^ water to 2 mg/mL. 2 µL of the diluted dispersions were drop-cast onto carbon-film coated TEM grids (Plano, Wetzlar, Germany). The excess dispersion was removed after 1 min with a filter paper. Preparation method 2 covers the room-temperature investigation of the cryo samples after the cryoTEM measurement. The cryo samples were kept under high vacuum while warming to room temperature slowly (i.e., freeze-dried) and subsequently examined. The micelles were no longer beam-sensitive in the freeze-dried state. Neither preparation method could avoid the formation of salt crystals when using salt solutions. The room-temperature TEM and cryoTEM micrographs shown here are representative of different locations on the TEM grids.

#### 2.3.6. Visual Turbidimetry

For a typical visual turbidimetry experiment, the P(DMA-*co*-NIPAM) copolymers were dissolved in MILLI-Q^®^ water and the 1% (*w*/*w*) dispersions were heated at a heating rate of approximately 1 C min^−1^. The temperature was monitored by a Greisinger GMH 3700^®^ Pt100 high precision thermometer (0.01 °C accuracy) (GHM Messtechnik GmbH, Regenstauf, Germany). Cloud points of the P(DMA-*co*-NIPAM) copolymers were estimated as the temperature at which turbidity first became visually apparent. In order to raise the accuracy, the measurement was repeated three times.

#### 2.3.7. Dynamic Light Scattering (DLS)

Temperature-dependent DLS measurements were conducted using an ALV^®^/CGS-3 Compact Goniometer-System (ALV-Laser Vertriebsgesellschaft m-b.H, Langen, Germany) employing an ALV^®^/LSE-5004 Multiple Tau Digital Correlator in combination with a Nd:YAG laser (532 nm, 400 mW). The temperature-dependent refractive indices and viscosities of water at each temperature were automatically corrected using the ALV^®^ Digital Correlator Software 3.0. The measurement angle was set to 90 °C for all 60 s measurements.

10 mg/mL (1% (*w*/*w*)) P(DMA-*co*-NIPAM) solutions were prepared by overnight stirring in pre-filtered MILLI-Q^®^ water to ensure complete dissolution. In order to investigate the effect of different anions on the thermoresponsive behavior, 0.5 M salt solutions were prepared beforehand and used to dissolve the polymer instead of pure MILLI-Q^®^ water. Each salt solution and the MILLI-Q^®^ water was pre-filtered through a microporous regenerated cellulose filter (average pore dia-meter of 200 nm) in a dust-free quartz glass vial. Before measurement, the samples were allowed to settle for at least 12 h to assure that any residual dust particles would settle and not interfere with the measurement. The quartz glass vial containing the 1% (*w*/*w*) copolymer solution was placed in the middle of a toluene bath used to avoid the laser light’s reflection and maintain the temperature. A Julabo F25 thermostat (Julabo GmbH, Seelbach, Germany) operating with a water/ethylene glycol mixture tempered the measuring cell filled with toluene with a temperature accuracy of 0.01 °C. The temperature ranges were set from approximately 10 °C below the cloud point (CP, previously determined via visual turbidimetry), to around 3 °C above the CP. Temperature-dependent DLS measurements were performed in temperature steps of 1 °C with two runs per temperature step. Temperature-dependent DLS measurements of the P(DMA-*co*-NIPAM)-*b*-PS diblock copolymers were performed in temperature steps of 1 °C with two runs per temperature step. The diblock copolymer dispersions were withdrawn directly after the polymerization from the polymerization mixture and diluted with pre-filtered MILLI-Q^®^ water to 0.2 mg/mL (targeted solution concentration 0.02% (*w/w*)). The temperature range was set from 25 °C to 45 °C. The evaluation of all DLS measurements was accomplished with a data evaluation software developed by F. Lauterbach [62]. The intensity correlation function ($g_{2}\left(\tau \right)$) was fitted with the function:

(1) $$g_{2}\left(\tau \right)=B+\beta \exp\left(-2\bar{\Gamma }\tau \right){\left(1+\frac{{µ}_{2}}{2!}\tau^{2}-\frac{{µ}_{3}}{3!}\tau^{3}+\cdots \right)}^{2}$$

where B represents the baseline, β denotes the stretching factor, $\bar{\Gamma }$ is the mean decay rate, ${µ}_{2}$, and ${µ}_{3}$ are the cumulants. The hydrodynamic radius $R_{H}$ was estimated from the first order cumulant via the Stokes-Einstein equation:

(2) $$R_{H}=\frac{k_{B}Tq^{2}}{6\pi \eta \bar{\Gamma }}$$

With $k_{B}$ the Boltzmann constant, T the temperature, q the scattering vector and η the viscosity.

## 3. Results and Discussion

### 3.1. Random Copolymerization of DMA and NIPAM

**Figure 1:**
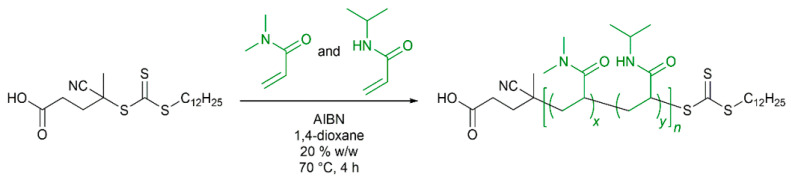
Synthesis of poly((*N,N*-dimethyl acrylamide)-*co*-(*N*-isopropyl acrylamide)) (P(DMA-*co*-NIPAM)) via RAFT solution polymerization in 1,4-dioxane (25% (*w/w*)).

**Figure 2:**
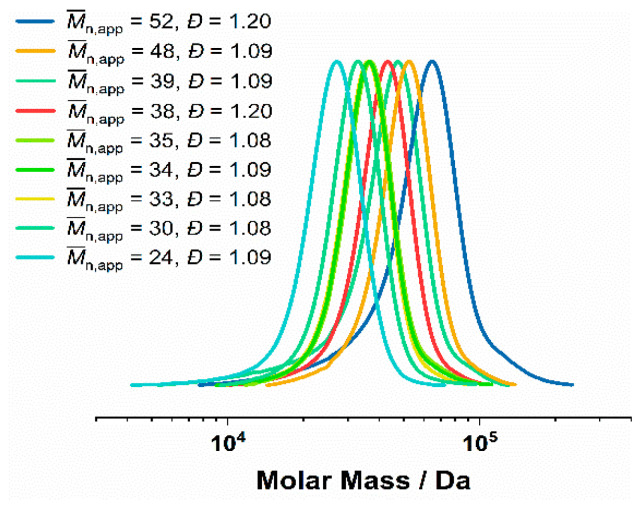
GPC of P(DMA-*co*-NIPAM) synthesized via RAFT solution polymerization in 1,4-dioxane (25% (*w*/*w*)). Dispersity of the respective GPC curve are shown in the legend.

A series of thermoresponsive P(NIPAM-*co*-DMA)-*b*-PS diblock copolymers were synthesized via RAFT emulsion polymerization. Within this series, the molecular weight fractions of NIPAM and DMA were varied, ranging from 99% NIPAM/1% DMA to 50% NIPAM/50% DMA. The P(NIPAM-*co*-DMA) copolymer precursors were prepared via RAFT solution polymerization, using 4-cyano-4-[(dodecylsulfanylthiocarbonyl)-sulfanyl]pentanoic acid (CDTPA) as CTA and very low amounts of AIBN as radical initiator (Figure 1). P(NIPAM-*co*-DMA) formed the mCTA for the subsequent RAFT emulsion polymerization of styrene. The monomer conversions of NIPAM and DMA were estimated via ^1^H NMR spectroscopy. In an exemplary polymerization, the conversion of DMA was calculated as 98.5% and the conversion of NIPAM as 94.0% after 4 h of polymerization. The polymerization was stopped after 4 h to maintain a high fidelity of the trithiocarbonate functionality and avoid the occurrence of an excess of termination products from coupling reactions [63,64]. The copolymers were characterized by GPC in DMAc as eluent (Figure 2). However, the obtained apparent molecular weight values should be handled with care due to both the calibration with polystyrene (different hydrodynamic volume of the polystyrene standard and the sample copolymer) and the possibility of interactions of the polar P(DMA-*co*-NIPAM) with the GRAM solid phase of the GPC column [65].

The P(DMA-*co*-NIPAM) GPC measurements show dispersities of *Đ* = 1.08–1.20 and apparent number average molecular weights (${\bar{M}}_{n,\mathrm{app}}$) of 24–52 kDa. The conversions of the two monomers, determined by ^1^H NMR, ranged from 90.0–99.9% for DMA and 78.0–97.0% for NIPAM. Overall, the GPC and ^1^H NMR results indicated well-controlled RAFT solution polymerizations at high conversions.

### 3.2. Determination of Copolymerization Parameters According to the Method of Kelen–Tüdős

The copolymer compositions were determined via ^1^H NMR spectroscopy. The molecular characteristics of the synthesized P(DMA-*co*-NIPAM) random copolymers and the molar DMA fractions of each copolymer are summarized in Table 1. In order to determine the copolymerization parameters of *N*-isopropyl acrylamide (NIPAM) and *N,N*-dimethyl acrylamide (DMA), the extended Kelen-Tüdős method was applied at high conversions [66]. The reactivity ratios were calculated as *r*_NIPAM_ = 0.83 and *r*_DMA_ = 1.10, respectively. These reactivity values agree with the values determined by Bauri et al. up to the second decimal place [61]. The ratios’ values are close to 1; therefore, it can be assumed that the copolymers are random/statistical copolymers since a given active chain end will non-selectively add the two monomers. The extended Kelen-Tüdős plot and parameters for the RAFT copolymerization of DMA and NIPAM in 1,4-Dioxane at 70 °C can be found in Table S1 and Figure S2.

### 3.3. Emulsion Polymerization of Styrene

**Figure 3:**
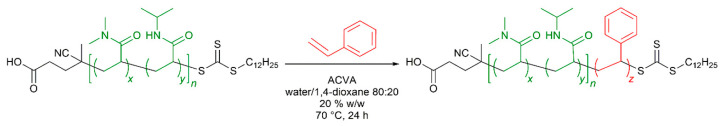
Synthetic route to poly((*N,N*-dimethyl acrylamide)-*co*-(*N*-isopropyl acrylamide))-*b*-polystyrene (P(DMA-*co*-NIPAM)-*b*-PS) diblock copolymers via surfactant-free RAFT emulsion polymerization in water/1,4-dioxane 80:20 (20% *w/w*).

The surfactant-free RAFT emulsion polymerization requires a well-dissolved mCTA to stabilize the formed latex and prevent coagulation [33]. This method of PISA via RAFT-mediated emulsion polymerization is characterized by the mCTA being soluble in the polymerization medium, but both the growing block and the monomer to be polymerized being insoluble [67]. However, thermoresponsive LCST-type mCTAs are generally unsuitable since they are usually insoluble at the usual RAFT polymerization temperatures of 65–70 °C. Nevertheless, in order to exploit the enormous advantages of RAFT emulsion polymerization for the synthesis of thermoresponsive styrenic block copolymers, such as fast reaction rates, low viscosities over the entire reaction period and final polymers exhibiting high molecular weights [33,37,68], a small amount of the suitable organic co-solvent 1,4-dioxane was added to the polymerization medium to increase the LCST above 70 °C [33]. The addition of a co-solvent makes the P(DMA-*co*-NIPAM) mCTA soluble at 70 °C in the aqueous polymerization medium. CDTPA-terminated P(NIPAM-*co*-DMA) acted as both the mCTA and macro-stabilizer in the emulsion polymerization of styrene, forming the desired P(NIPAM_x_-*co*-DMA_y_)-*b*-PS_z_ diblock copolymers with x, y, and z being the mean numbers of the respective monomer units of P(NIPAM-*co*-DMA) and PS, respectively. An 80:20 water/1,4-dioxane mixture was selected as the continuous phase for these RAFT emulsion polymerization formulations. The water-soluble 4,4′-azobis(4-cyanopentanoic acid) (ACVA) was used to initiate the polymerization (Figure 3).

### 3.4. ^1^H NMR and GPC Characterization of the P(DMA-co-NIPAM)-b-PS Copolymers

**Figure 4:**
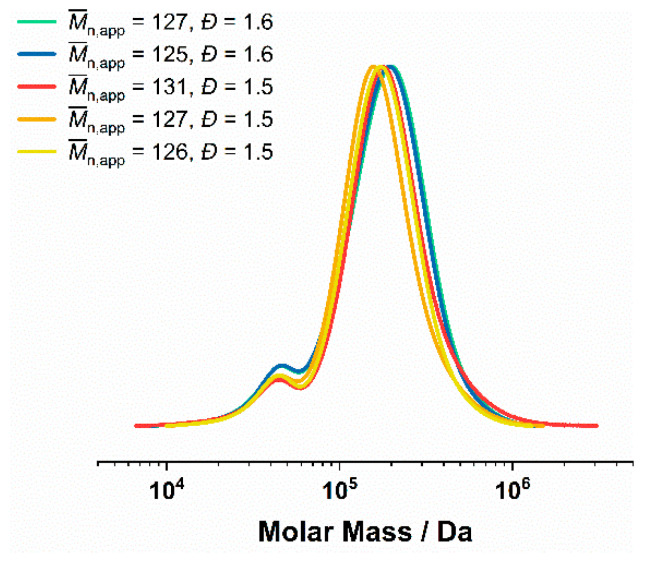
GPC of P(DMA-*co*-NIPAM)-*b*-PS synthesized via RAFT emulsion polymerization in water/1,4-dioxane 80:20 (20% *w*/*w*) using either P(DMA_148_-NIPAM_127_)^29^ or P(DMA_199_-NIPAM_171_)^39^ as mCTA. ${\bar{M}}_{n,\mathrm{app}}$ (determined by DMAc GPC calibrated with PS standards) and dispersity of the respective GPC curve are shown in the legend. Impurities due to residues of the random copolymer (mCTA P(DMA_148_-NIPAM_127_) or P(DMA_199_-NIPAM_171_)) can be detected in the GPC traces.

**Figure 5:**
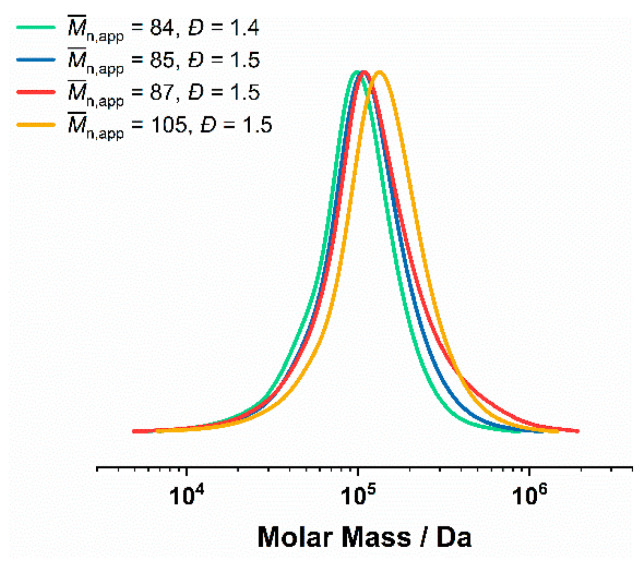
GPC of P(DMA-*co*-NIPAM)-*b*-PS synthesized via RAFT emulsion polymerization in water/1,4-dioxane 80:20 (20% *w/w*) using either the P(DMA_29_-NIPAM_180_)^24^ or P(DMA_35_-NIPAM_212_)^28^ as mCTA. The ${\bar{M}}_{n,\mathrm{app}}$ (determined by DMAc GPC calibrated with PS standards) and the dispersity of the respective GPC curve are shown in the legend.

^1^H NMR revealed a quantitative monomer conversion in the emulsion polymerization of styrene within a polymerization time of 24 h. The apparent number average molecular weights (${\bar{M}}_{n,\mathrm{app}}$) of the P(DMA-*co*-NIPAM)-*b*-PS diblock copolymers were obtained by DMAc GPC and the corresponding exemplary GPC curves are shown in Figure 4 and Figure 5. The dispersities of *Đ* = 1.4–1.6 taken from these GPC curves indicate controlled RAFT emulsion polymerizations.

Nevertheless, impurities due to residues of the random copolymer (mCTA P(DMA_148_-NIPAM_127_) or P(DMA_199_-NIPAM_171_)) can be detected in the GPC traces (Figure 4), accounting for about 12–16% of the total polymer. They can probably be assigned to unreacted mCTA chains. However, quantitative evaluation of the chromatograms shows acceptable dispersities (*Đ* = 1.5–1.6) of high molecular weight diblock copolymers due to high styrene conversions of up to 98%. The weight fraction of the minor P(DMA-*co*-NIPAM) block in the diblock copolymers was calculated by ^1^H NMR at 23–30 wt.%.

When other precursors (mCTA P(DMA_29_-NIPAM_180_)^24^ or P(DMA_35_-NIPAM_212_)^28^) were used for the emulsion polymerization of styrene, hardly any unreacted mCTA residues were visible in the GPC traces (Figure 5). In this case, dispersities of 1.4–1.5, high molecular weights between 121–141 kDa (${\bar{M}}_{n,\mathrm{th}}$) and P(DMA-*co*-NIPAM) weight fractions of 17–20 wt.% could be obtained.

To complete the initial characterization of the diblock copolymers, the chosen method of aqueous RAFT emulsion polymerization is suitable to prepare high molecular weight diblock copolymers under simple reaction conditions in acceptable reaction times and yields low viscosities over the entire reaction period. This very robust synthetic route, which does not require surfactants or other additives, enables the preparation of P(DMA-*co*-NIPAM)-*b*-PS diblock copolymers with high molecular weights above 120 kDa (${\bar{M}}_{n,\mathrm{app}}$) due to high styrene conversions of 66–98%. Table 2 summarizes the molecular characteristics of the obtained diblock copolymers.

### 3.5. Morphology of Diblock Copolymer Thin Films

As the diblock copolymers prepared by the PISA process are potential candidates for applications in filtration membranes or temperature-sensitive coatings, their bulk state was investigated in order to characterize the polymers more comprehensively. Transmission electron microscopy (TEM) measurements were performed on the P(DMA-*co*-NIPAM)-*b*-PS diblock copolymer films obtained by solution casting from CHCl_3_.

**Figure 6:**
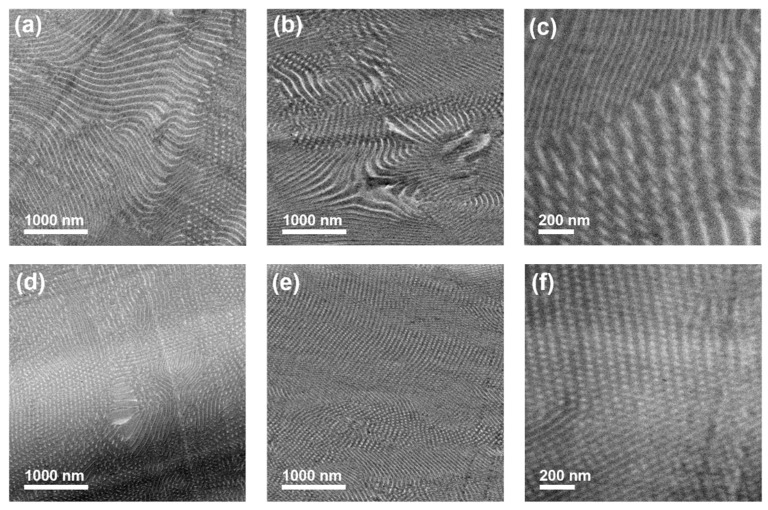
TEM images of the P(DMA-*co*-NIPAM)-*b*-PS bulk films. (**b**,**c**,**e**,**f**) The PS phase was selectively stained in RuO_4_ vapor to increase the contrast. The PS phase appears dark and the P(DMA-*co*-NIPAM) phase bright. (**a**) P(DMA_29_-NIPAM_180_)-PS_937_^121^ unstained bulk film; (**b**,**c**) P(DMA_29_-NIPAM_180_)-PS_937_^121^ stained bulk film; (**d**) P(DMA_29_-NIPAM_180_)-PS_944_^122^ unstained bulk film; (**e**,**f**) P(DMA_29_-NIPAM_180_)-PS_944_^122^ stained bulk film.

Figure 6 shows TEM images of the P(DMA_29_-NIPAM_180_)-PS_937_^121^ and P(DMA_29_-NIPAM_180_)-PS_944_^122^ diblock copolymer films. Since the P(DMA-*co*-NIPAM) block is water-soluble, dry-sectioning was chosen for all films, which resulted in creases on the film’s surface and lower contrast. The PS matrix was selectively stained with RuO_4_.

Concerning a P(DMA_29_-NIPAM_180_)-PS_937_^121^ sample with a 121 kDa and a P(DMA-*co*-NIPAM) weight fraction of 20%, the cylindrical morphology was identified, with bright lines of P(DMA-*co*-NIPAM) cylinders in a continuous, dark PS matrix oriented parallel to the plane, while domains of bright dots indicate perpendicularly oriented P(DMA-*co*-NIPAM) cylinders in the PS matrix (Figure 6a–c). The cylindrical morphology was also observed for P(DMA_29_-NIPAM_180_)-PS_944_^122^ with a 122 kDa and a P(DMA-*co*-NIPAM) weight fraction of 19%. However, compared to the previous sample, larger sections of perpendicularly aligned cylinders can be seen for P(DMA_29_-NIPAM_180_)-PS_944_^122^ in Figure 6d–f). Parallel aligned P(DMA-*co*-NIPAM) cylinders were observed in particular in Figure 6e and partially in Figure 6d,f).

### 3.6. Solubility Study of P(DMA-co-NIPAM) Random Copolymers and P(DMA-co-NIPAM)-b-PS Diblock Copolymers in Water

#### 3.6.1. Cloud Point Measurement via Visual Turbidimetry

In order to investigate the solubility behavior of a temperature-responsive polymer, turbidimetry measurement usually permits a good indication of the cloud point. It is a first insight into the solubility behavior of the LCST-type polymer but does not provide any information on temperature-dependent hydrodynamic radii evolution. In order to determine the hydrodynamic radii and their evolution and investigate the solubility behavior in more detail, DLS measurements were carried out after the cloud point determination via visual turbidimetry.

Cloud points of the P(DMA-*co*-NIPAM) copolymers were estimated as the temperature at which turbidity first became visually apparent (Table 3). As expected, an increase in the molar fraction of the more hydrophilic DMA leads to an increase of the copolymers’ LCST. This increase was almost linearly dependent on the molar fraction of DMA in the copolymer. The samples were stirred during the heating and cooling cycles to ensure a homogeneous temperature distribution. Almost no hysteresis was observed, which could be due to stirring and the relative insensitivity of the method. This phenomenon will be discussed in more detail in the context of DLS measurements.

Thus, the results are in agreement with literature where hydrophilic/hydrophobic systems with different copolymer compositions were studied and dependence of the LCST on the composition could be ascertained. Taylor and Cerankowski claimed that the LCST of thermoresponsive copolymers could be continuously varied by incorporating a more hydrophilic component [69]. This statement has since been validated by the behavior of a number of hydrophilic/hydrophobic copolymer systems [55,70,71,72].

#### 3.6.2. DLS Measurements

**Figure 7:**
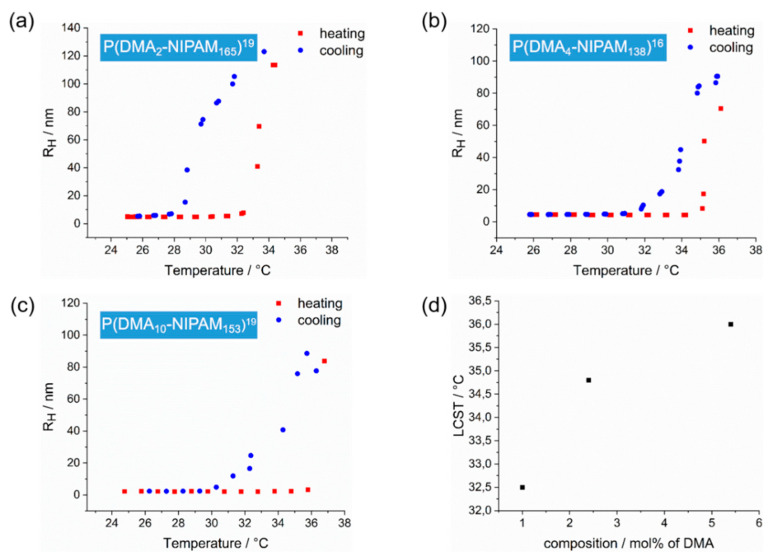
Temperature-dependent evolution of the hydrodynamic radii of three P(DMA-*co*-NIPAM) copolymers with different copolymer compositions in aqueous solution (c = 1% (*w*/*w*)) obtained by DLS. The red dots show the evolution of the hydrodynamic radii during the heating step of the polymer solution. In contrast, the blue dots represent the development of the corresponding radii during the cooling step. Hydrodynamic radii evolution of (**a**) P(DMA_2_-NIPAM_165_)^19^, (**b**) P(DMA_4_-NIPAM_138_)^16^, and (**c**) P(DMA_10_-NIPAM_153_)^19^. (**d**) LCST versus copolymer composition. The LCST increases with the increasing molar fraction of DMA.

Temperature-dependent DLS measurements allow for a detailed investigation of the changes in the hydrodynamic radii of the thermoresponsive copolymers and thus of their solution behavior (Figure 7).

Immediately striking is the hysteresis in the solubility behavior of the copolymers in the heating and cooling cycles, which occurs due to the delay in the dissolution of the large aggregated and precipitated particles.

A strong hysteresis can be explained by the nature of DLS procedures as well as by the chemical nature of PNIPAM. DLS measurements exclude stirring during the measurement. Thus, large precipitated particles take longer to redissolve. As the dissolution behavior of PNIPAM is a result of its intramolecular and intermolecular interactions, in the globular state strong intrachain hydrogen bonding prevents, according to Wang et al., rapid rehydration and thus leads to a pronounced hysteresis [73].

Since the LCST is also molecular weight dependent, copolymers with almost the same molecular weight of 16 kDa and 19 kDa, respectively, as determined by ^1^H NMR, and dispersity were selected to compare the solubility behavior of random copolymers with different molar DMA fractions. The LCST for the random copolymer with a molar DMA fraction of 1% (32.5 °C) is almost 2 °C higher than the LCST of the copolymer with a DMA content of 2.4% (34.8 °C). Accordingly, for the copolymer with the highest molar DMA fraction of 5.4%, the highest LCST of 36 °C is observed. Thus, there is a noticeable increase in LCST as a function of the molar fraction of the unrestricted water-soluble DMA. The most pronounced hysteresis is observed for the copolymer with the smallest DMA content and thus the largest NIPAM content of 98.9%. This is probably due to the strong intrachain bonding of PNIPAM described by Wang et al. [73]. In comparison with Table 3, it can also be noted that there is a respective difference of approximately 2 °C between the LCSTs determined by DLS and the cloud points determined by visual turbidimetry. This occurs since the samples were stirred during the turbidimetry measurement, whereas DLS measurements take place without any form of stirring.

#### 3.6.3. Effect of Additives

The following part of this study discusses the influence of additives on the thermoresponsiveness of the copolymer solutions. Today’s research on the thermodynamics of the influence of anions and cations on the solubility of macromolecules are based on the results of Franz Hofmeister [74,75,76]. An influence on the solubility behavior of thermoresponsive polymers was recognized, especially for anions [11,77]. In the so-called Hofmeister series, the chaotropic, that is the structure-breaking effect of the anions, increases from left to right. The (kosmotropic) anions further to the left enhance the hydrophobic effects in aqueous protein solutions and thus promote protein aggregations via hydrophobic interactions (salting-out). This favors the precipitation of proteins. The (chaotropic) anions further to the right increase the solubility of hydrophobic molecules in water (salting-in):

$${\mathrm{CO}}_{3}{}^{2-}>{\mathrm{SO}}_{4}{}^{2-}>H_{2}{\mathrm{PO}}_{4}{}^{-}>F^{-}>{\mathrm{Cl}}^{-}>{\mathrm{Br}}^{-}\approx {\mathrm{NO}}_{3}{}^{-}>I^{-}>{\mathrm{ClO}}_{4}{}^{-}>{\mathrm{SCN}}^{-}$$

Sodium salts of these anions were used to investigate the influence of different anions on the solubility behavior of the P(DMA-*co*-NIPAM) random copolymers. The anions in bold were used for this study.

**Figure 8:**
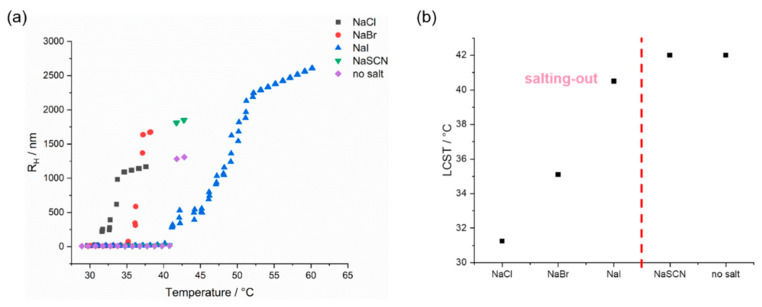
(**a**) The effect of anions on the hydrodynamic radii evolution of P(DMA_35_-NIPAM_212_)^28^ (c = 1% (*w*/*w*)) as a function of temperature calculated by DLS measurements. (**b**) The impact of the anions on the LSCT of P(DMA_35_-NIPAM_212_)^28^ determined by DLS measurements. The concentration of the sodium salt solutions was 0.5 M in all measurements.

The solubility behavior of a P(DMA_35_-NIPAM_212_)^28^ random copolymer dissolved in MILLI-Q^®^ water or the 0.5 M salt solutions of the anions listed above was investigated by DLS, and the changes in hydrodynamic radii are shown in Figure 8. Since the copolymer dissolved in the 0.5 M salt solutions precipitates above LCST and does not readily re-dissolve, not least due to the absence of stirring during the DLS experiment, only the heating cycles are shown here. Among the anions of the Hofmeister series employed here, Cl^−^ and Br^−^ showed a significant salting-out effect, as they lowered the LCST, while I^−^ caused only a slight decrease in the LCST and SCN^−^ exerted no influence on the LCST. Thus, the anions Cl^−^ and Br^−^ showed the expected kosmotropic effects, while the anions I^−^and SCN^−^ had little to no effect on the LCST. The transition between salting-out and salting-in effect for other LCST-type homo- and copolymers that have been studied, was found between Cl^−^ and Br^−^ or Br^−^ and I^−^, respectively [11,77]. In the case of the aqueous random copolymer solutions studied in this work, the transition between salting-out and salting-in effect seems to have shifted further to the right along with the Hofmeister series, towards the intrinsically chaotropic salts [74].

Presumably, the salting-out effect is related to the high surface charge density of the kosmotropic anions. This increases the surface tension in the inner hydration shell of the polymer. Kosmotropic salts, therefore, exhibit a strongly negative hydration entropy [78,79]. In other words, in the presence of kosmotropic anions, fewer water molecules are available for the hydration of the polymer. Thus, the LCST decreases. In addition, hydrophobic-hydrophobic interactions are enhanced by the higher polarity of the solvent in the presence of salt. In the case of chaotropic anions, Zhang et al. found for PNIPAM that the salting-in effect decreases with increasing molecular weight and polymer concentration [80]. For PNIPAM with a molecular weight of =31 kDa and dissolved in a 0.5 M NaSCN or NaI solution (c = 1% (*w*/*w*)), only a weak salting-in effect was observed. They attributed the findings, showing that higher polymer concentrations, as well as higher molecular weight, that cause a less pronounced salting-in effect, to the suggestion that the dependence on molecular weight is caused by the increased intramolecular interactions, while the concentration effect is caused by increased intermolecular interactions. In other words, an increase in polymer concentration or molecular weight leads to more pronounced chain-chain interactions. In the case of the P(DMA_35_-NIPAM_212_)^28^ random copolymer studied here, which has a comparable molecular weight and was investigated at the same polymer concentration (c = 1% (*w*/*w*), there may be similarly pronounced chain-chain interactions so that the intrinsically chaotropic anions SCN^−^ and I^−^ have no or even a weak salting-out effect.

#### 3.6.4. Aqueous Dispersions of the P(DMA-*co*-NIPAM)-*b*-PS Micelles

**Figure 9:**
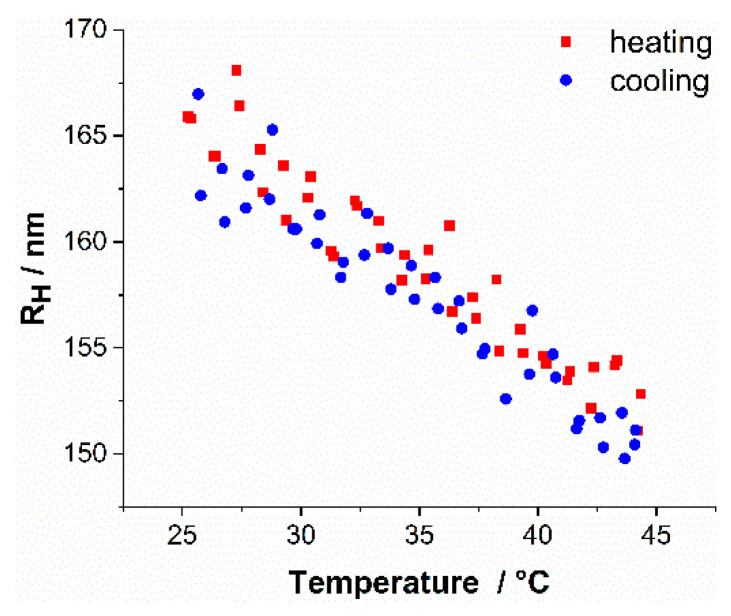
Hydrodynamic radii evolution of P(DMA_35_-NIPAM_212_)-PS_1089_^141^ (c = 0.02% (*w*/*w*)) as a function of temperature determined by DLS measurements. The red dots represent the hydrodynamic radii during the heating of the polymer dispersion and the blue dots represent the corresponding hydrodynamic radii during the cooling step. The LCST of the copolymer precursor P(DMA_35_-NIPAM_212_)^28^ is 42 °C, as determined by DLS measurements.

An essential part of this study was to examine the solubility behavior of a P(DMA-*co*-NIPAM) block if it is bound to an insoluble block such as PS. More specifically, the aim was to evaluate whether the confinement leads to a change in the cloud point or swelling behavior of the P(DMA-*co*-NIPAM) block compared to the free chain without PS block. Thus, a P(DMA-*co*-NIPAM)-*b*-PS diblock copolymer in aqueous systems was studied as a function of temperature. For this purpose, diluted samples (0.02% (*w*/*w*) solids concentration) of the P(DMA-*co*-NIPAM)-*b*-PS diblock copolymer micelles were analyzed using DLS in a temperature range of 25–45 °C. The original polymer dispersion was diluted with MILLI-Q^®^ water. In addition to water, the original polymer dispersion also contained the co-solvent 1,4-dioxane. Thus, also the diluted samples retained a 1,4-dioxane content of 0.016% (*w/w*). The development of the hydrodynamic radii is shown for P(DMA_35_-NIPAM_212_)-PS_1089_^141^ in Figure 9.

Analysis of the DLS data revealed that the dispersions consist of only one particle species in the range of 150–168 nm (at 25 to 45 °C). Accordingly, all polymer chains are aggregated into micelles and there are no unimers left in the solution. When the particle dispersion is heated, the hydrodynamic radius of the micelles decreases as a nearly linear function of temperature. Towards the end of the heating ramp, between 40 and 45 °C, a slight reduction in the decrease was observed. This radius decrease can be attributed to the collapse of the P(DMA-*co*-NIPAM)-corona block. It is completely reversible until the aggregate size reaches its initial value at 25 °C. Coagulations were not observed.

The heating and cooling cycles were fitted, showing an equal slope for both, indicating a hysteresis-free, completely reversible change in micelle size. An explanation for the gradual size change that the micellar aggregates undergo upon heating, in contrast to the sharp phase transition of the random copolymer precursor, is that the densely packed P(DMA-*co*-NIPAM) chains at the surface of the micelle prevent the water molecules from penetrating the entire corona. By heating the aqueous micelle dispersions, the originally dense-packed hydrated P(DMA-*co*-NIPAM) corona blocks are gradually dehydrated, gradually collapsing, leading to a decrease in the hydrodynamic radius of the micelles. The gradual decrease in radius is also promoted by the fact that the P(DMA-*co*-NIPAM) blocks cannot collapse freely. The decrease in micelle size is 10% of the original size, which is within the size range of the weight fraction of the thermoresponsive P(DMA_35_-NIPAM_212_) block of 20%. This leads to the assumption that by heating further above 45 °C, a reduction of the radius would have been quite possible. Since this temperature-dependent behavior is rather progressive and shows no hysteresis, the phrase “thermosensitive” is used in the terminology instead of “thermoresponsive” [33,81].

#### 3.6.5. Influence of Salt on Micellar Assemblies

**Figure 10:**
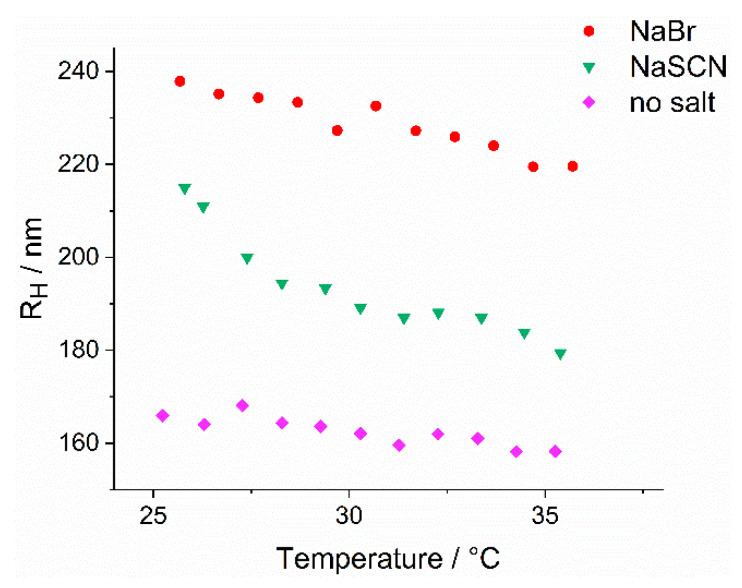
The effect of NaBr or NaSCN on the hydrodynamic radii evolution of P(DMA_35_-NIPAM_212_)-PS_1089_^141^ (c = 0.02% (*w*/*w*)) as a function of temperature determined by DLS measurements. The micellar dispersions were heated from 25 °C to 35 °C. The concentration of the sodium salt solutions was 0.5 M in all measurements.

The previous part of the study has revealed that PS-bound thermoresponsive copo-lymers behave differently in water than as free chains. The next point under investigation is how the previously discussed Hofmeister-salts influence the behavior of the micelles compared to the free chains. As salt reactivity, or the amplification of existing responsivities by salts, is an interesting and potentially useful tuning feature of micellar assemblies [82], the contribution of salts to the temperature-induced collapse of the micelle corona was investigated. The response of P(DMA-*co*-NIPAM)-*b*-PS micelles to salts was exemplarily evaluated by the addition of NaBr and NaSCN, respectively, to the aqueous micellar dispersions (Figure 10).

Figure 10 shows that the addition of Br^−^ at 25 °C leads to an almost 1.5-fold increase in the hydrodynamic radius compared to the hydrodynamic radius of the aqueous micellar solution. The addition of SCN^−^ at 25 °C also resulted in a 1.3-fold increase in the hydrodynamic radius to nearly 220 nm. It is also striking that adding these anions to the aqueous micellar solution leads to a much faster collapse of the micelles’ corona when heated to 35 °C. A fit of the heating cycles yielded a negative slope of the hydrodynamic radius twice as large for Br^−^ and even a negative slope four times as large for SCN^−^ compared to the negative slope of the aqueous micellar solution. When NaSCN is added to the aqueous micelle dispersions, the hydrodynamic radius decreases rapidly by a total of 17% when heated from 25 °C to 35 °C. In comparison, the hydrodynamic radius decreases in the same temperature range by 8% in a 0.5 M solution of NaBr and by only 4% in the pure aqueous micellar solution. Again, in contrast to the sharp phase transition of the random copolymer precursors, the explanation for the gradual size change undergone by the micellar aggregates upon heating is probably the dense packing of the P(DMA-*co*-NIPAM) chains at the surface of the micelle. This dense packing impedes the penetration of the water molecules and thus also of the salt anions, dissolved in water, into the entire corona. However, due to the addition of salt, the water molecules presumably penetrate somewhat further into the corona due to the relatively greater elongation, resulting in less densely packed chains. This also leads to more severe dehydration during heating of the micelles and thus to a more pronounced decrease in micelle size. During the investigation of the influence of the salt anions on the P(DMA-*co*-NIPAM) mCTA, no influence on the LCST was found for SCN^−^, while Br^−^ had a more significant effect on the LCST. In the case of the diblock copolymer, the influence of SCN^−^ on the radius of the micelles also appears to be less than that of Br^−^. Since the diameter of the micelle core is not affected by the addition of salts, Br^−^ seems to penetrate more effectively into the corona, leading to a more significant increase in the hydrodynamic radius, and is also repelled more slowly from the corona upon an increase in temperature.

To conclude, the micelles can be influenced in their size by the addition of suitable additives such as salts, and moreover, their thermosensitivity can be strengthened and accelerated by the addition of salt anions.

#### 3.6.6. Morphological Study of the P(DMA-*co*-NIPAM)-*b*-PS Micelles

**Figure 11:**
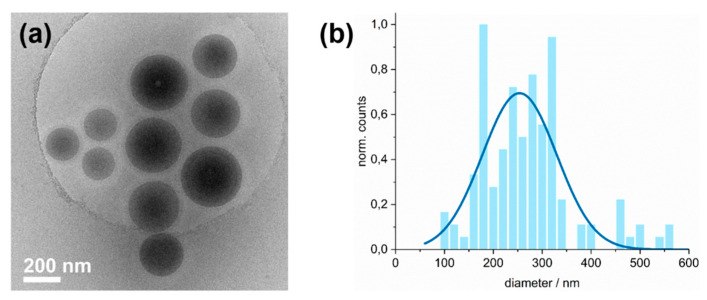
(**a**) Exemplary cryoTEM micrograph of the diluted P(DMA_35_-NIPAM_212_)-PS_1089_^141^ dispersion. Only the dense micelle core is visible in the micrographs due to the low contrast of the swollen corona against amorphous ice. The polymer dispersion was diluted with MILLI-Q^®^ water to obtain a final latex concentration of c = 0.2% (*w*/*w*). (**b**) The histogram shows the size distribution of latex particles taken from the analysis of cryoTEM micrographs of the sample. A Gaussian fit (blue line) revealed a mean diameter of 254 ± 152 nm. For the analogous room-temperature TEM micrograph and size distribution, see Figure S3.

Once the thermosensitive nature of the nanoparticles was confirmed, the diluted polymer dispersion was further investigated using cryo and room-temperature TEM to accurately determine the morphology of the micelles and also to visualize their shape and size. As described before, the samples were directly withdrawn from the dispersions after polymerization and diluted with MILLI-Q^®^ water to obtain final polymer concentrations of 2 and 0.2 mg/mL. With the low contrast of the swollen P(DMA-*co*-NIPAM) corona against amorphous ice, only the dense PS core of the micelles is visible in the cryoTEM images (Figure 11). Therefore, it is hard to make accurate size interpretation for the entire synthesized block copolymer. However, both a theoretical minimum value of the diameter of the entire micelle (corona-chain in theta state) and a theoretical maximum value of the micelle diameter (corona-chain fully stretched (all-trans conformation)) were calculated. The minimum micelle diameter was calculated using the radius of gyration, which in turn is calculated from the number of monomer units, the number of bonds per monomer unit, the segmental length, and the polymer characteristic ratio [83,84]. Thus, for the minimum micelle diameter, a value of 272 nm is obtained. The fully stretched length of the corona chain was calculated using the contour length and corresponds to an extreme that can hardly be reached. For the maximum micelle diameter, a value of 378 nm is obtained. The value determined by DLS was 168 nm at 25 °C. The experimentally determined micelle diameter, therefore, lies between the two calculated values. The interpretation that individual micelles and not aggregated micelles predominate can therefore be assumed to be correct.

The cryoTEM images show that all P(DMA_35_-NIPAM_212_)-corona PS_1089_^141^-core micelles generated with PISA are spherical (Figure 11). Micelle morphologies other than spherical, such as wormlike micelles or vesicles, were not encountered. This observation agrees well with literature reports stating that PS, as the hydrophobic core of the micelle, has little ability to reorganize from spherical micelles to worm-like aggregates due to its high glass transition temperature [37,85].

Additionally, the cryoTEM images indicate a fraction of large, presumably superswollen micelles, whose involvement in the polymerization process could be a reason for the sample’s molecular weight distribution of 1.4, which is not entirely narrow. As the concentration of mCTA and thus the radical concentration is very low during polymerization compared to the styrene concentration, the superswelling of the micelles could occur faster than their nucleation and the growth of the PS block and thus proceed partly preferentially [33,86]. Moreover, it is likely that the rather long stabilizing P(DMA-*co*-NIPAM) block of 28 kDa prevents the spherical micelles from fusing and forming wormlike or higher-order morphologies, even when the solid content of 20% was in the range (i.e., 10% to 25%) [87] that should facilitate morphology transformation [85,88]. On the other hand, an additional parameter that could assist in this observation is that during the emulsion polymerization process, the size of the particles is not in an absolute equilibrium attaining only one size.

**Figure 12:**
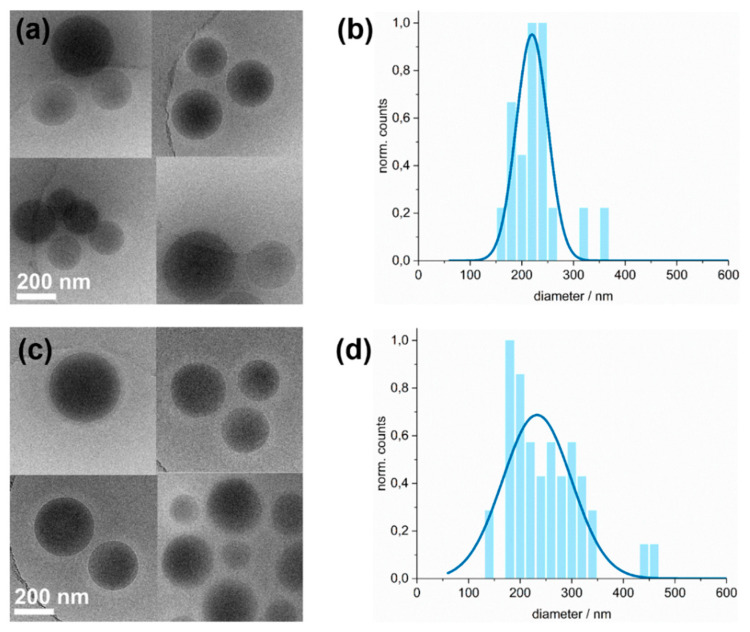
CryoTEM micrographs and evaluation of the particle size distributions of P(DMA_35_-NIPAM_212_)-PS_1089_^141^ dispersions diluted with 0.5 M salt solutions to a final latex concentration of c = 0.2% (*w*/*w*). Due to the low contrast of the swollen corona against amorphous ice, only the dense micelle core is visible in the micrographs. (**a**) Exemplary cryoTEM micrographs of a P(DMA_35_-NIPAM_212_)-PS_1089_^141^ dispersion diluted with an aqueous 0.5 M NaBr solution, (**b**) the histogram shows the size distribution of latex particles taken from analysis of cryoTEM micrographs of the sample exemplary shown in (**a**). A Gaussian fit (blue line) gave a mean diameter of 220 ± 61 nm; (**c**) Exemplary cryoTEM micrographs of a P(DMA_35_-NIPAM_212_)-PS_1089_^141^ dispersion diluted with an aqueous 0.5 M NaSCN solution, (**d**) size distribution of latex particles taken from analysis of cryoTEM micrographs of the sample exemplary shown in (**c**). The histogram shows the size distribution of the micelles. The mean diameter of 233 ± 133 nm was determined by a Gaussian fit. For the analogous TEM micrographs at room temperature and size distributions of the samples diluted with the aqueous salt solutions, see Figure S4.

Confirmation that the cryoTEM images show only the glassy core of the micelles is provided by the cryoTEM images of the polymer dispersions diluted with aqueous 0.5 M salt solutions. Figure 12 shows the P(DMA_35_-NIPAM_212_)-PS_1089_^141^ polymer dispersions, which were diluted with 0.5 M NaBr or NaSCN solutions to 2 mg/mL. DLS measurements showed that the micelles in the samples diluted with 0.5 M NaBr or 0.5 M NaSCN solutions, respectively, exhibited larger hydrodynamic radii than those diluted with MILLI-Q^®^ water. These larger R_H_ can most likely be attributed to a corona with more elongated chains. In cryoTEM, however, where the swollen corona is invisible due to its low contrast, another effect can be observed. The core of the micelle seems to have a slightly smaller diameter in the latex samples diluted with the aqueous salt solutions. One possible explanation might be that the more polar salt solution forces the hydrophobic PS core to become more densely packed. However, the clarification of this assumption was beyond the scope of this work.

The micelles’ mean diameter, determined by a Gaussian fit, is in very good agreement with the R_H_ value of 168 nm determined by DLS (at 25 °C). The mean diameter of the PS core was determined by cryoTEM to be 254 nm. Accordingly, the combination of the DLS and cryoTEM results leads to the estimation that the PS core is 24% smaller than the entire micelle including the corona. Since P(DMA-*co*-NIPAM) chains form the corona of the micelle and a P(DMA-*co*-NIPAM) content of 20 wt.% was determined by ^1^H NMR, these values fit very well.

Room-temperature TEM micrographs were acquired for comparison with the cryoTEM micrographs (Figures S3 and S4). It was found that the mean diameters of the micelle-cores as determined by Gaussian fits, were insignificantly larger for cryoTEM than for room-temperature TEM. This applies to both the dispersion diluted with MILLI-Q^®^ water (Figure S3) and the dispersions diluted with the aqueous NaBr and NaSCN solutions (Figure S4), respectively. To be more precise, in room-temperature TEM images, mean micellar diameters of 248 ± 117 nm were found for P(DMA_35_-NIPAM_212_)-PS_1089_^141^ dispersions diluted with MILLI-Q^®^ water, 213 ± 61 nm for the sample diluted with 0.5 M NaBr solution, and 221 ± 105 nm for the sample diluted with NaSCN solution (for corresponding TEM images, histograms and Gaussian fits, see Figures S3 and S4). For the dispersion diluted with MILLI-Q^®^ water, the dispersion diluted with a 0.5 M NaBr solution and the dispersion diluted with a 0.5 M NaSCN solution, an increase in the mean micellar diameter of 2%, 3% and 5%, respectively, was observed. Since the room-temperature TEM and cryoTEM samples have undergone the same sample preparation, with the exception of plunging the cryoTEM samples into liquid ethane, the latter must lead to a slight swelling of the core of the micelle, insofar as this can be judged from the data.

To conclude, well-defined spherical micelles with a glassy PS core and a thermosensitive P(DMA-*co*-NIPAM) corona can be conveniently generated using RAFT-mediated PISA, as evaluated using DLS and cryoTEM data. Our results are in agreement with fin-dings for other styrenic copolymers containing a thermoresponsive minor block and for which a fully reversible temperature-induced corona collapse has been observed [33,37].

## 4. Conclusions

This work presents a two-step solution–emulsion polymerization of P(DMA-*co*-NIPAM)-*b*-PS diblock copolymers. In the first step, P(DMA-*co*-NIPAM) random copo-lymers were synthesized via RAFT solution polymerization. The incorporation of DMA into the polymer structure increases the LCST as a function of DMA content. The effect of salt anions on the solubility behavior of the random copolymers was studied. Cl^−^ and Br^−^ showed kosmotropic effects, while I^−^ and SCN^−^ had little to no impact on the LCST in aqueous solutions. In the second synthesis step, high molecular weight P(DMA-*co*-NIPAM)-*b*-PS diblock copolymers were prepared via surfactant-free RAFT emulsion polymerization of styrene. CryoTEM proved that the P(DMA_35_-NIPAM_212_)-corona PS_1089_-core micelles generated via PISA were spherical. The confinement by the glassy PS core drastically changed the temperature-dependent swelling behavior of the P(DMA-*co*-NIPAM) corona chains. While a sharp phase transition was observed for the thermoresponsive free P(DMA-*co*-NIPAM) chains, a gradual collapse of the corona was observed for the chains bound to the insoluble PS core. The addition of Br^−^ or SCN^−^ to the aqueous micellar dispersions increased the hydrodynamic radius of the micelles at 25 °C. Moreover, adding these anions to the aqueous micellar solution resulted in a faster collapse of the micelle corona when heated to 35 °C.

As the diblock copolymers prepared by the PISA process are potential candidates for applications in filtration membranes or temperature-sensitive coatings, their bulk state was investigated. TEM thin film investigation revealed that the diblock copolymers microphase-separated into a regularly ordered hexagonal cylindrical morphology, with the thermoresponsive minor P(DMA-*co*-NIPAM) block forming cylinders and the major PS block forming the matrix.

## Figures and Tables

**Table 1:** Molecular characteristics of the synthesized P(DMA-*co*-NIPAM) random copolymers, listing the molar DMA fractions of each copolymer.

Copolymer ^a^	${{\bar{M}}_{n,th}}^{b}\;[\mathrm{kDa}]$	${{\bar{M}}_{n,app}}^{c}\;[\mathrm{kDa}]$	Conv._DMA_ [%]	Conv._NIPAM_ [%]	Number of DMA Units ^d^	Number of NIPAM Units ^d^	*Đ*	*f*_DMA feed_ [%]	*f*_DMA copolymer_ [%]
P(DMA_148_-NIPAM_127_)^29^	29	38	99.0	97.0	148	127	1.20	50	50.5
P(DMA_199_-NIPAM_171_)^39^	39	52	99.6	97.0	199	171	1.20	50	50.5
P(DMA_43_-NIPAM_69_)^13^	13	18	90.0	78.0	43	69	1.08	30	33.2
P(DMA_29_-NIPAM_180_)^24^	24	39	98.5	94.0	29	180	1.09	12	12.5
P(DMA_35_-NIPAM_212_)^28^	28	48	98.0	92.0	35	212	1.09	12	12.7
P(DMA_10_-NIPAM_153_)^19^	19	33	99.9	93.0	10	153	1.08	5	5.4
P(DMA_6_-NIPAM_160_)^19^	19	34	99.9	95.5	6	160	1.08	3	3.1
P(DMA_4_-NIPAM_138_)^16^	16	30	95.0	82.0	4	138	1.09	2	2.4
P(DMA_2_-NIPAM_165_)^19^	19	38	99.9	96.0	2	165	1.08	1	1.1

^a^ Subscripts denote the mean numbers of the respective monomer units as determined by ^1^H NMR spectroscopy. Superscripts represent the total estimated molecular weight. ^b^ was calculated as follows: ^c^ Determined by DMAc GPC calibrated with PS standards. ^d^ Denote the mean numbers of the respective monomer units.

**Table 2:** Molecular characteristics of the synthesized P(DMA-*co*-NIPAM)-*b*-PS diblock copolymers, listing the respective weight fraction of the PS block.

Diblock Copolymer ^a^	${{\bar{M}}_{n,th}}^{b}\;[\mathrm{kDa}]$	${{\bar{M}}_{n,app}}^{c}\;[\mathrm{kDa}]$	Conv. [%]	*Đ*	DP_PS_^d^	*f*_P(DMA-*co*-NIPAM)_ [wt.%]
P(DMA_148_-NIPAM_127_)-PS_887_^122^	122	127	98	1.6	887	23
P(DMA_148_-NIPAM_127_)-PS_1024_^136^	136	125	96	1.6	1024	26
P(DMA_148_-NIPAM_127_)-PS_663_^98^	98	131	66	1.5	663	30
P(DMA_148_-NIPAM_127_)-PS_8381_^116^	116	127	98	1.5	838	25
P(DMA_148_-NIPAM_127_)-PS_794_^112^	112	126	98	1.5	794	26
P(DMA_29_-NIPAM_180_)-PS_937_^121^	121	84	71	1.4	937	20
P(DMA_29_-NIPAM_180_)-PS_944_^122^	122	87	76	1.5	944	19
P(DMA_35_-NIPAM_212_)-PS_1125_^141^	141	85	79	1.5	1125	17
P(DMA_35_-NIPAM_212_)-PS_982_^130^	130	105	82	1.5	982	21
P(DMA_35_-NIPAM_212_)-PS_1089_^141^	141	129	84	1.4	1089	20
P(DMA_29_-NIPAM_180_)-PS_1120_^140^	140	111	91	1.4	1120	17

^a^ Subscripts denote the mean numbers of the respective monomer units as determined by ^1^H NMR spectroscopy. Superscripts represent the total. ^b^ was calculated as follows: ^c^ determined by DMAc GPC calibrated with PS standards. ^d^ Denotes the mean degree of polymerization of polystyrene (DP).

**Table 3:** Cloud points of P(DMA-*co*-NIPAM) random copolymers as obtained via visual turbidimetry.

Copolymer	CP [°C] ^a^	*f* _PDMA Copolymer_ [%]
P(DMA_2_-NIPAM_165_)^19^	34.5	1.1
P(DMA_4_-NIPAM_138_)^16^	36.6	2.4
P(DMA_10_-NIPAM_153_)^19^	38.8	5.4
P(DMA_35_-NIPAM_212_)^28^	42.5	12.7

^a^ Cloud points of the P(DMA-*co*-NIPAM) copolymers were estimated as the temperature at which turbidity first became visually apparent.

## Data Availability

The characterization data are available upon request from the authors.

## Supplementary Materials

The following are available online at https://www.mdpi.com/article/10.3390/polym14010062/s1, Figure S1: ^1^H NMR spectra recorded in (a) CDCl_3_ for a P(DMA_29_-NIPAM_180_)^24^ macroRAFT agent; (b) in THF-*d*_8_ for a P(DMA_29_-NIPAM_180_)-PS_944_^122^ diblock copolymer. Table S1: Extended Kelen-Tüdős parameters for the RAFT copolymerization of DMA and NIPAM in 1,4-Dioxane at 70 °C; Figure S2: Determination of *r*_DMA_ and *r*_NIPAM_ by the extended Kelen-Tüdős method; Figure S3: TEM image and evaluation of the particle size distributions of P(DMA_35_-NIPAM_212_)-PS_1089_^141^. (a) Exemplary TEM micrograph of a diluted P(DMA_35_-NIPAM_212_)-PS_1089_^141^ dispersion in water and at room temperature (preparation method 1). (b) Size distribution of latex particles taken from analysis of TEM micrographs of the sample exemplary shown in (a). Figure S4 TEM images and evaluation of the particle size distributions of P(DMA_35_-NIPAM_212_)-PS_1089_^141^ dispersions diluted with 0.5 M salt solutions. (a) Exemplary TEM micrographs of a P(DMA_35_-NIPAM_212_)-PS_1089_^141^ dispersion diluted with an aqueous 0.5 M NaBr solution, freeze-dried and at room temperature (preparation method 2). (b) Size distribution of latex particles taken from analysis of TEM micrographs of the sample exemplary shown in (a). (c) Exemplary TEM micrograph of a P(DMA_35_-NIPAM_212_)-PS_1089_^141^ dispersion diluted with an aqueous 0.5 M NaSCN solution and freeze-dried at room temperature (preparation method 2). (d) Size distribution of latex particles taken from analysis of TEM micrographs of the sample exemplary shown in (c).
