# Supplementary material for: RAFT Emulsion Polymerization of Styrene Using a Poly((N,N-dimethyl acrylamide)-co-(N-isopropyl acrylamide)) mCTA: Synthesis and Thermosensitivity
Source: Polymers (Basel). 2021 Dec 24;14(1):62. doi: 10.3390/polym14010062 (PMC8747436; doi:10.3390/polym14010062)
Supplement: Supplementary file 1 [file polymers-14-00062-s001.zip › polymers-1523606-supplementary.pdf]

Supplementary information for:

RAFT emulsion polymerization of styrene using a poly((*N,N*-dimethyl acrylamide)-*co*-(*N*-isopropyl acrylamide)) mCTA: synthesis and thermosensitivity

Katharina Nieswandt[1], Prokopios Georgopanos[1]*, Martin Held[1], Evgeni Sperling[1], and Volker Abetz[1,2,]*

[1] Helmholtz-Zentrum Hereon, Institute of Membrane Research, Max-Planck-Straße 1, 21502 Geesthacht, Germany; katharina.nieswandt@hereon.de, prokopios.georgopanos@hereon.de, martin.held@hereon.de, evgeni.sperling@hereon.de, volker.abetz@hereon.de

[2] Institute of Physical Chemistry, University of Hamburg, Martin-Luther-King-Platz 6, 20146 Hamburg, Germany;

***** Correspondence: prokopios.georgopanos@hereon.de, volker.abetz@hereon.de

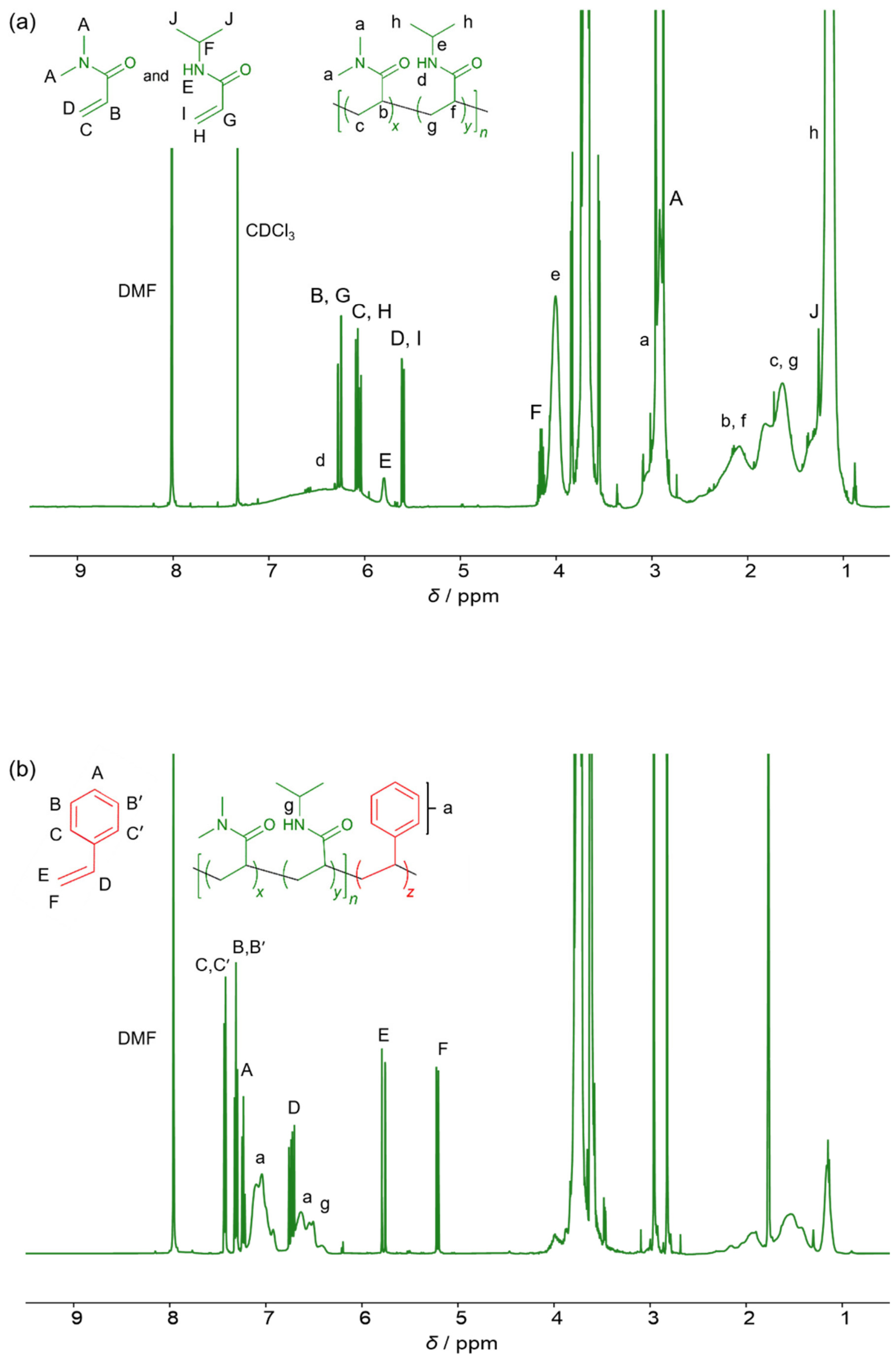

Figure S1. ^{1}H NMR spectra recorded in (a) $CDCl_3$ for a P(DMA$_{29}$-NIPAM$_{180}$)[24] macroRAFT agent; (b) in THF-d_8 for a P(DMA$_{29}$-NIPAM$_{180}$)-PS$_{944}$[122] diblock copolymer.

Table S1. Extended Kelen-Tüdős parameters for the RAFT copolymerization of DMA and NIPAM in 1,4-Dioxane at 70 °C. The extended Kelen-Tüdős method is applicable for the calculation of reactivity ratios at high conversion. Several parameters (G, H, η, μ) were determined using the equations bellow table S1. The reactivity ratios were determined from the extended Kelen-Tüdős plot (Fig. S2).

$f_{\text{DMA feed}}$	$F_{\text{DMA copolymer}}$	G	H	H	μ
0.500	0.510	0.0392	0.9608	0.0360	0.8828
0.300	0.330	-0.3673	0.3411	-0.7838	0.7278
0.120	0.130	-0.8095	0.0169	-5.6021	0.1172
0.020	0.024	-0.7762	0.1244	-3.0801	0.4938

The parameters where determined using the following equations:

$$G = \frac{f_{\text{DMA feed}}\left(2F_{\text{DMA copolymer}} - 1\right)}{(1 - f_{\text{DMA feed}})F_{\text{DMA copolymer}}} \quad (1)$$

$$H = \frac{{f_{\text{DMA feed}}}^{2}\left(1 - F_{\text{DMA copolymer}}\right)}{(1 - f_{\text{DMA feed}})^{2}F_{\text{DMA copolymer}}} \quad (2)$$

The arbitrary constant α is introduced, with where H_{min} and H_{max} are the lowest and highest values of H:

$$\alpha = (H_{\text{min}}H_{\text{max}})^{0.5} \quad (3)$$

$$\eta = \frac{G}{(\alpha + H)} \quad (4)$$

$$\mu = \frac{H}{(\alpha + H)} \quad (5)$$

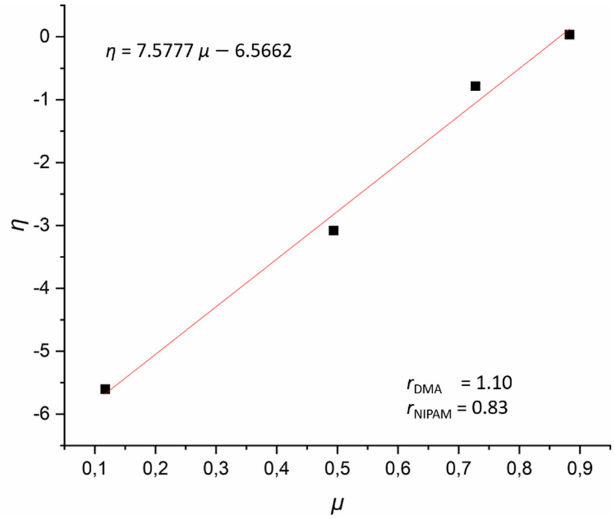

Figure S2. Determination of r_{DMA} and r_{NIPAM} by the extended Kelen-Tüdős method. The four data points represent four different polymerizations with different fractions of DMA in the feed. The calculated parameters are withdrawn from table S1. The reactivity ratios were determined using equation (3) and (6) and the Kelen-Tüdős plot.

$$\eta = \left(r_{NIPAM}\frac{r_{DMA}}{\alpha}\right)\mu - \frac{r_{DMA}}{\alpha} \tag{6}$$

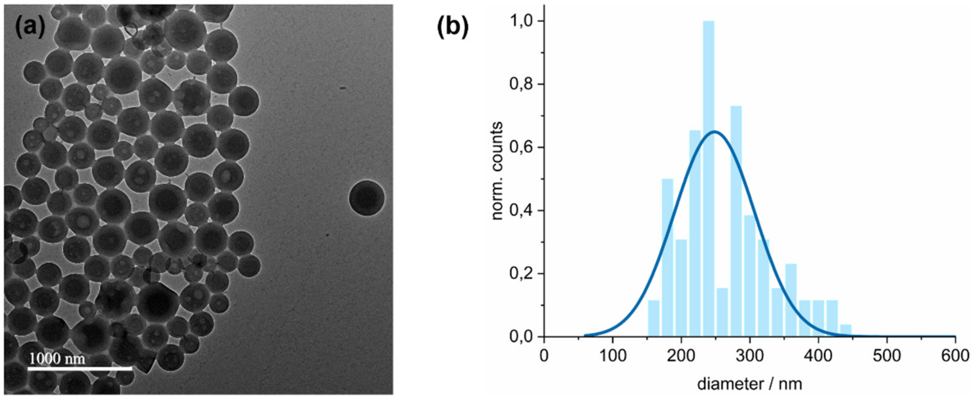

Figure S3. TEM image and evaluation of the particle size distributions of P(DMA_{35}-$NIPAM_{212}$)-PS_{1089}^{141}. (a) Exemplary TEM micrograph of a diluted P(DMA_{35}-$NIPAM_{212}$)-PS_{1089}^{141} dispersion in water and at room temperature (preparation method 1). The polymer dispersion was diluted with MILLI-Q® water to obtain a final polymer concentration of c = 0.2 % (w/w). (b) Size distribution of latex particles taken from analysis of TEM micrographs of the sample exemplary shown in (a). The histogram shows the size distribution of the micelles. A Gaussian fit (blue line) gave a mean diameter of 248 ± 117 nm.

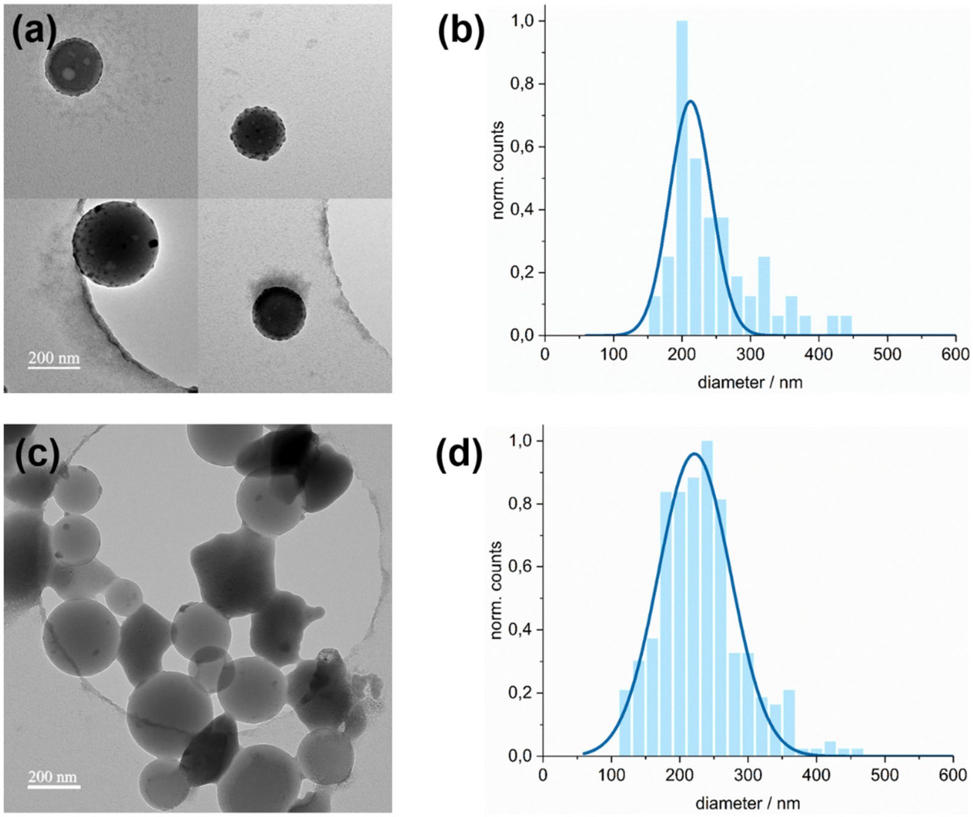

Figure S4. TEM images and evaluation of the particle size distributions of P(DMA_{35}-$NIPAM_{212}$)-PS_{1089}[141] dispersions diluted with 0.5 M salt solutions to a final concentration of c = 0.2 % (w/w). (a) Exemplary TEM micrographs of a P(DMA_{35}-$NIPAM_{212}$)-PS_{1089}[141] dispersion diluted with an aqueous 0.5 M NaBr solution, freeze-dried and at room temperature (preparation method 2). (b) Size distribution of latex particles taken from analysis of TEM micrographs of the sample exemplary shown in (a). The histogram shows the size distribution of the micelles. A Gaussian fit (blue line) gave a mean diameter of 213 ± 61 nm; (c) Exemplary TEM micrograph of a P(DMA_{35}-$NIPAM_{212}$)-PS_{1089}[141] dispersion diluted with an aqueous 0.5 M NaSCN solution and freeze-dried at room temperature (preparation method 2). (d) Size distribution of latex particles taken from analysis of TEM micrographs of the sample exemplary shown in (c). The histogram shows the size distribution of the micelles. The mean diameter of 221 ± 105 nm was determined by a Gaussian fit.
